# Association of TERT *(rs2736098 and rs2736100)* genetic variants with elevated risk of hepatocellular carcinoma: a retrospective case–control study

**DOI:** 10.1038/s41598-023-45716-w

**Published:** 2023-10-26

**Authors:** Walaa R. Seif Eldin, Entsar A. Saad, Ahmed Monier, Rami M. Elshazli

**Affiliations:** 1 Department of Chemistry, Faculty of Science, Damietta University, Damietta, 34517 Egypt; 2 Department of Digestive Surgery, Faculty of Medicine, Mansoura University, Mansoura, Egypt; 3 Biochemistry and Molecular Genetics Unit, Department of Basic Sciences, Faculty of Physical Therapy, Horus University – Egypt, New Damietta, 34518 Egypt

**Keywords:** Biochemistry, Biological techniques, Chemical biology, Computational biology and bioinformatics, Genetics, Molecular biology

## Abstract

Hepatocellular carcinoma (HCC) is an inflammatory problematic issue with higher mortality among different ethnic populations. The telomerase reverse transcriptase (*TERT*) gene has an imperative role in the proliferation of various cancerous illnesses, particularly HCC. Moreover, the *TERT (rs2736098 and rs2739100)* variants were correlated with the HCC susceptibility and telomere shortening, but with unconvincing outcomes. The main purpose of this outward work is to assess the correlation between these significant variants within the *TERT gene* and the elevated risk of HCC with the aid of various computational bioinformatics tools. This study included 233 participants [125 cancer-free controls and 108 HCC patients] from the same locality. In addition, 81.5% of HCC patients were positive for HCV autoantibodies, while 73.1% of HCC patients were positive for cirrhotic liver. Genomic DNA of the *TERT (rs2736098 and rs2736100)* variants were characterized utilizing the PCR–RFLP method. Interestingly, the frequencies of TERT (rs2736098*A allele) and TERT (rs2736100*T allele) conferred a significant correlation with increased risk of HCC compared to healthy controls (*p*-value = 0.002, and 0.016, respectively). The TERT (rs2736098*A/A) genotype indicated a definite association with positive smoking and splenomegaly (*p*-value < 0.05), while the TERT (rs2736100*T/T) genotype observed a significant difference with higher levels of HCV autoantibodies (*p*-value = 0.009). In conclusion, this significant work confirmed the contribution of the TERT (rs2736098*A and rs2736100*T) alleles with elevated risk of HCC progression and telomere shortening among Egyptian subjects.

## Introduction

**Figure 1:**
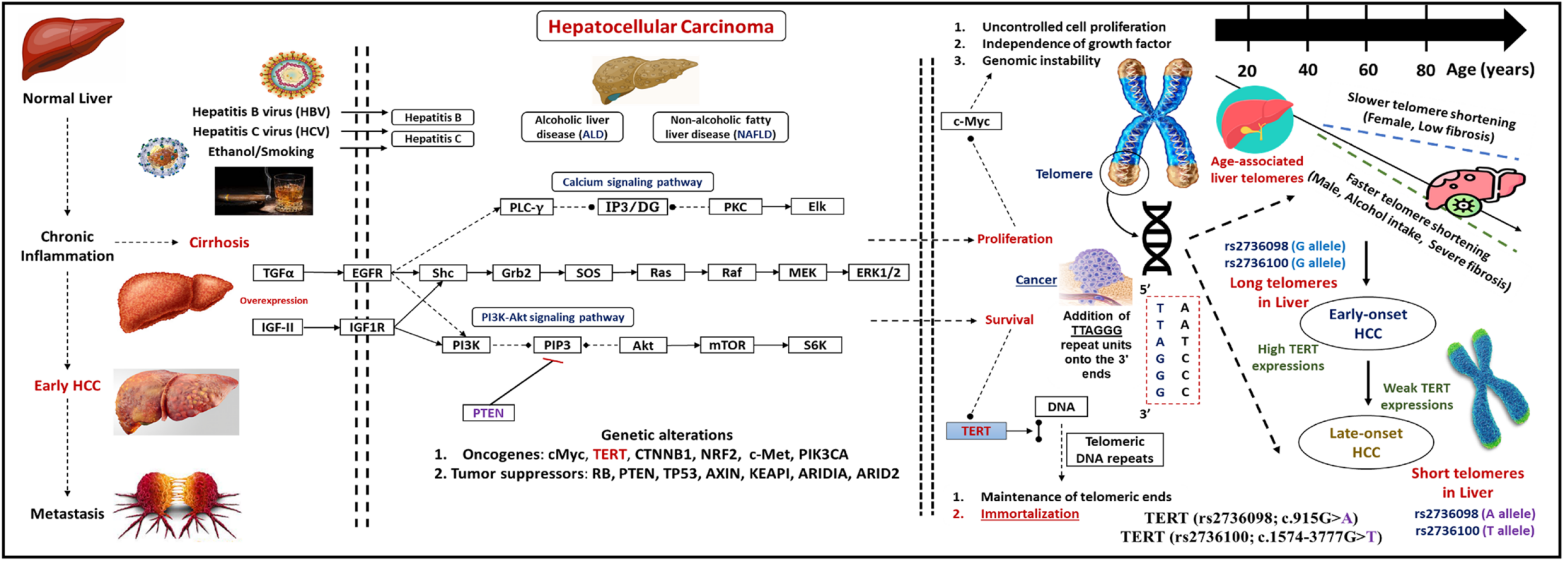
The possible pathogenesis mechanisms for the HCC development with telomere length. The *TERT gene* encoded a ribonuclease telomerase enzyme that maintained the chromosomal ends from attrition by the addition of repetitive nucleotide units (TTAGGG) at the 3′ prime ends of the genome. The immortal cancerous tissues in the hepatocytes prevent cellular apoptosis by increasing the telomerase expression at early-onset HCC and decreasing the telomerase expression at late-onset HCC and thus enhancing the telomere shortening. The *TERT (rs2736098; A allele)* and *TERT (rs2736100; T allele)* were associated with telomere shortening and increased risk of hepatocellular carcinoma. Abbreviations: HCC, Hepatocellular carcinoma; TERT, telomerase reverse transcriptase.

Hepatic cancer is the seventh incidence cancer distributed worldwide occupying about 8.7% of cancer-related deaths with higher mortality recorded within developing countries^1,2^. Universally, hepatocellular carcinoma (HCC) is a global issue that requires a particular strategy to decline the mortality percentages associated with elevated incidence of this type of carcinoma^3,4^. The awareness of the molecular basis of HCC development is essential to recognize the pathogenesis mechanisms that correlate with the adverse effects of this type of hepatic-associated cancer, (Fig. 1)^5,6^. In Egypt, HCC is the second cancer distributed locally compared to other carcinomas, with incidence rates of 20.7% of the total cancerous patients identified among Egyptian subjects^2,7^. In addition, numerous reports with technological improvements indicated the potential impact of genetic background on the pathogenesis of hepatic carcinogenesis^8–10^.

The human telomerase reverse transcriptase (*TERT*) gene has a crucial function in the proliferation of different numbers of cancerous illnesses, particularly hepatic carcinoma^6,11,12^. The *TERT* gene (OMIM#: 187270, other synonyms include TRT, TP2, hEST2, and EST2) is located along the reverse strand on chromosome 5p15.33 and comprised of seven splice variants with the main transcript (TERT-201; ENST00000310581.10) that included 16 exons and 15 introns encoding a powerful ribonuclease enzyme which is referred as telomerase with 1132 amino acids^13–15^. This ribonuclease telomerase enzyme is a multicomplex protein with two main parts: telomerase reverse transcriptase (TERT) along with its core template, telomerase RNA component^16,17^. Furthermore, the telomerase has a significant function in the maintenance of the integrity of small structural elements at the terminal of the chromosomes that are called telomeres through the addition of repetitive nucleotide units (TTAGGG) at the 3′ prime ends of the genome^18,19^. These terminal sequences of telomeres have starring roles in the capping of the chromosomal terminus to prevent fusion and recombination of the chromosomes^8,20^.

Moreover, the attrition of these repeated units through cell cycle, genome replication, and aging could increase the levels of genomic instability, cell arresting, and apoptosis^21,22^. Nevertheless, the immortal cancerous tissues could overwhelm this inevitable fate by stimulating the expression rate of telomerase to lengthen the telomere structures at the chromosomal terminus^16,23^. Two potential genetic variants within the *TERT gene* involving TERT*(rs2736098; c.915G > *A)* and TERT*(rs2736100; c.1574-3777G > *T)* have a critical role in the elevated risk of several cancerous disorders including rectal cancer^24^, renal cell carcinoma^19^, prostate cancer^25^, and hepatocellular carcinoma^26–28^, but with inconclusive conclusions.

Intriguingly, hepatic cancer is considered a hyperinflammatory problem with adverse complications that could lead to cellular apoptosis and genomic variations ^5,8^. During the late-onset HCC, the immortal tumor tissues could decrease the telomerase activity within the hepatic tissues leading to telomere shortening and liver cirrhosis ^29,30^. Moreover, limited reports clarified the contributions of the synonymous TERT*(rs2736098*A allele) and the intron TERT*(rs2736100*T allele) variants with telomere shortening and increased risk of HCC progression among different ethnic populations ^6,26,31^. Due to the limited data concerning the role of the *TERT gene* in the progression of HCC, our team was motivated to design this research to estimate the association of these significant variants within the *TERT gene* and the HCC susceptibility among Egyptian subjects with the practice of various computational bioinformatics tools.

## Materials and methods

### Ethical statement approval

This work was authorized and achieved an approval announcement from the ethical committee board [IRB #: R.22.10.1899] at the Faculty of Medicine, Mansoura University, Egypt. In addition, the study protocol commenced according to the declaration guidelines of Helsinki recommendations. All enrolled subjects were asked to allocate an informed written consent upon participation in this work.

### Study participants

This retrospective case–control study included a sum of 233 participants: comprising 108 HCC patients [81.5% males, and 18.5% females], together with 125 unrelated cancer-free controls [73.6% males, and 26.4% females] that matched with age, gender, and geographical district. All HCC patients were collected from the Oncology Clinics at Gastrointestinal Surgery Center, Mansoura University, Egypt, as formerly described^5^. The diagnostic criteria for the selection of HCC patients were executed based on imaging techniques involving computerized tomography (CT) and/or magnetic resonance imaging (MRI)^32^. Furthermore, HCC patients who experienced previous history of malignancies, autoimmune disorders, hepatic problems, renal diseases, diabetic mellitus, alcohol abuse, metabolic diseases, and/or other endocrine issues were omitted from this research. The clinical and demographic characteristics were extracted from medical archives.

### Sample collection and genomic DNA extraction

Under complete aseptic conditions, a total of 10 ml of venous blood was gathered and obtained from all participants in this study. The blood samples were sectioned into two aliquots; the first portion was collected in a vacutainer tube containing EDTA anticoagulant for hematological and genetic purposes, while the subsequent portion was processed within serum separator tubes and subjected to centrifugation to separate serum aliquot for biochemical and serological measurements. Furthermore, the identification of biochemical assessments was performed using Roche Cobas c501 biochemical analyzer (Roche Diagnostics, Manheim, Germany), while the evaluation of serological examinations was carried out utilizing ADVIA Centaur® CP Immunoassay system (Siemens Healthineers, Germany) including alpha-fetoprotein (AFP), and hepatitis C virus autoantibodies (Anti-HCV). Additionally, the estimation of hematological parameters was substantiated using Abbott CELL-DYN 3700 SL hematology analyzer (Abbott Diagnostics, USA)^33^.

### Genomic DNA extraction and purification

The manipulation of genomic DNA from peripheral EDTA blood was extracted utilizing Mini kit GeneJET Whole Blood Genomic DNA (Thermo Fischer Scientific, Waltham, MA, USA) according to the manufacturer’s instructions. The degree of purity of extracted genomic DNA was evaluated utilizing the NanoDrop™ ND-1000 Spectrophotometer technology^34^.

### Amplification of the TERT*(rs2736098; c.915G > *A)* variant

The genotyping of the TERT*(rs2736098; c.915G > A) and TERT*(rs2736100; c.1574-3777G > *T)* variants were executed using polymerase chain reaction with restriction fragment length polymorphism (PCR–RFLP) through pertaining enzymatic cleavage technique^35,36^. For the TERT*(rs2736098; c.915G > *A)* variant, the two primers used were [forward primer (F1): 5′- AGG ACG CGT GGA CCG AGT GAC-3′, and reverse primer (R1): 5′-GGA ACC CAG AAA GAT GGT CTC-3′, respectively]^36^. The PCR protocol was processed within a thermal cycler in total volume of 28 µl comprising of 4 µl of template genomic DNA, 4 µl of forward primer, 4 µl of reverse primer, 12.5 µl of 2X PCR Master Mix, 3.5 µl nuclease-free sterile water (ddH_2_O). The reaction conditions were computed with an initial denaturation step at 95 °C for 5 min, followed by 30 repetitive cycles of 95 °C for 30 s, 67 °C for 30 s, and 72 °C for 28 s, and a last extension step at 72 °C for 10 min. The amplified product of the TERT*(rs2736098; c.915G > *A)* variant generated a 324-bp fragment that was subjected to endonuclease cleavage by a restriction enzyme *Bsp120I/PspOMI* (New England Biolabs, Ipswich, MA, USA) at 37 °C for 60 min and could identify a cutting site (G/GGCCC). The G allele was digested and produced two fragments at 137-bp and 187-bp, while the undigested A allele generated a single band at 324-bp, (Figure S1)^36^.

### Amplification of the TERT*(rs2736100; c.1574-3777G > *T) *variant

Alternatively, the two primers used in the amplification of TERT*(rs2736100; c.1574-3777G > *T)* variant were [forward primer (F2): 5′-GCT GTT TTC CCT GCT GAC TT-3′, and reverse primer (R2): 5′-AGA ACC ACG CAA AGG ACA AG-3′, respectively] ^35^. Moreover, the reaction protocol was handled in a total volume of 30 µl comprising 4 µl of template genomic DNA, 4 µl of forward primer, 4 µl of reverse primer, 12.5 µl of 2X PCR Master Mix, 5.5 µl nuclease-free sterile water (ddH_2_O). The PCR conditions were adjusted with an initial denaturation step at 94 °C for 5 min, followed by 35 repetitive cycles of 94 °C for 30 s, 59.5 °C for 30 s, and 72 °C for 30 s, and a last extension step at 72 °C for 7 min. The amplified fragment for the TERT*(rs2736100; c.1574-3777G > *T)* variant produced a 194-bp band that is exposed to endonuclease cleavage by a restriction enzyme *SfcI* (New England Biolabs, Ipswich, MA, USA) at 37 °C for 60 min and could distinguish a cutting site (C/TRYAG). The digested fragments could generate two fragments for the wildtype G allele at 101-bp and 93-bp, while the rare T allele remained undigested with a single fragment at 194-bp, (Figure S2)^35^. The digested fragments were electrophoresed utilizing 2.5% agarose gel including 0.5 μg/ml ethidium bromide to facilitate the photographing process with UV illumination. Additionally, about one-tenth of the samples were independently subjected to repeat genotyping to achieve a degree of quality assurance under strict blind conditions and the corresponding results were acceptable with a percentage of 100%.

### Statistical analysis

In the beginning, the manipulation and tabulation of data extracted from this survey were accomplished using Stata Statistical Software, Release #17 (StataCorp. 2021, College Station, TX: LLC). The calculation of sample size of this presented work was computed utilizing the G*power software version 3.1.9.7, suggesting that the observed power (1–β error probability) attained a value of 82% with an effect size of 0.24^33^. The Shapiro–Wilk test was managed to assess the normality of the quantitative variables that were presented as median (IQR) for the nonparametric data. The allelic/genotypic distributions of the TERT*(rs2736098 and rs2736100) variants were computed utilizing Fisher's exact and Chi-squared test, as formerly described^37^. The web-established tool SNP analysis was conducted for the calculation of the Odds ratios (OR) and 95% confidence interval (CI) for the genetic models and haplotypic frequencies of the TERT*(rs2736098 and rs2736100) variants (www.snpstats.net/start.htm; accessed on 27 July 2022)^38^. According to the distribution of data types, the recessive model of the TERT (rs2736098*A/A and rs2736100*T/T) variants was analyzed with different clinical and laboratory parameters using Chi-square and two-sample Wilcoxon rank-sum tests. Moreover, the multivariate analysis including principal component analysis and correlation matrix was accomplished with the aid of R programming language software version 4.2.0 with R studio version 2022.07.1 Build 554^39^, while the meta-analysis design was established using Stata Statistical Software, Release #17. The level of statistical analysis was considered significantly beneficial with *p*-value < 0.05.

### Ethical approval

All procedures performed in studies involving human participants were subjected to following the ethical standards of the institutional research committee and with the 1964 Helsinki Declaration and its later amendments.

## Results

### The fundamental characteristics of the study population

This study included 233 participants that clustered into two subgroups comprising 108 HCC patients with a median age (IQR) was 53.0 (45.0–61.5) years, together with 125 cancer-free controls from the same locality with a median age (IQR) was 54.0 (49.0–58.0) years. There was no remarkable correlation among HCC patients compared to control subjects regarding age, gender, consanguinity, family history, cirrhotic liver, hypertension, ascites status, and splenomegaly (*p*-value > 0.05). Interestingly, HCC patients with positive smoking and high weights revealed a statistically significant compared with control subjects (*p*-value < 0.05). Based on viral infection, 88 HCC patients (81.5%) were positive for HCV autoantibodies, while 79 HCC patients (73.1%) were positive due to cirrhotic issues. Furthermore, higher levels of ALT, AST, total bilirubin, INR, AFP, and HCV autoantibodies were observed among HCC patients compared with control subjects (*p*-value < 0.001). Absolutely not, lower concentrations of albumin, RBCs, WBCs, hemoglobin, hematocrit, and platelets count were noticed among HCC patients compared with control subjects (*p*-value < 0.001) (Table 1).

**Table 1:** The main demographic, clinical, and laboratory parameters of the study participants.

Characteristic		Cancer-free controls	HCC patients	*P*-value
		(n = 125)	(n = 108)	
I. Demographic and clinical characteristics				
1. Age, years	Median (IQR)	54.0 (49.0–58.0)	53.0 (45.0–61.5)	0.865
2. Age groups (≤ 40/ > 40)	n (%)/n (%)	15 (12.0)/110 (88.0)	21 (19.4)/87 (80.6)	0.146
3. Weight, kg	Median (IQR)	80.0 (76.0–87.0)	84.0 (78.0–89.0)	**0.008**
4. Gender (Male/Female)	n (%)/n (%)	92 (73.6)/33 (26.4)	88 (81.5)/20 (18.5)	0.162
5. Smoking (Positive/Negative)	n (%)/n (%)	17 (13.6)/108 (86.4)	32 (29.6)/76 (70.4)	**0.004**
6. Consanguinity (Positive/Negative)	n (%)/n (%)	**–**	26 (24.1)/82 (75.9)	NA
7. Family history (Positive/Negative)	n (%)/n (%)	**–**	19 (17.6)/89 (82.4)	NA
8. Cirrhotic liver (Positive/Negative)	n (%)/n (%)	**–**	79 (73.1)/29 (26.9)	NA
9. Hypertension (Positive/Negative)	n (%)/n (%)	**–**	33 (30.6)/75 (69.4)	NA
10. Ascites status (Presence/Absence)	n (%)/n (%)	**–**	72 (66.7)/36 (33.3)	NA
11. Splenomegaly (Presence/Absence)	n (%)/n (%)	**–**	94 (87.0)/14 (13.0)	NA
II. Biochemical measurements				
1. ALT, U/L	Median (IQR)	29.0 (21.0–35.0)	51.1 (33.0–84.5)	** < 0.001**
2. AST, U/L	Median (IQR)	26.0 (21.0–31.0)	64.0 (42.0–112.5)	** < 0.001**
3. Albumin, g/l	Median (IQR)	42.0 (39.0–47.0)	31.0 (27.0–35.5)	** < 0.001**
4. Total bilirubin, mg/dl	Median (IQR)	0.90 (0.70–1.00)	1.60 (1.10–3.30)	** < 0.001**
5. Direct bilirubin, mg/dl	Median (IQR)	0.21 (0.16–0.26)	0.84 (0.48–2.12)	** < 0.001**
6. Indirect bilirubin, mg/dl	Median (IQR)	0.67 (0.51–0.84)	0.81 (0.55–1.28)	** < 0.001**
7. International normalized ratio (INR)	Median (IQR)	1.00 (1.00–1.10)	1.20 (1.10–1.40)	** < 0.001**
8. Creatinine, mg/dl	Median (IQR)	0.90 (0.70–1.00)	0.98 (0.80–1.30)	** < 0.001**
III. Serological investigations and Tumor markers				
1. Anti-HCV (Positive/Negative)	n (%)/n (%)	0.0 (0.0)/125 (100.0)	88 (81.5)/20 (18.5)	** < 0.001**
2. AFP, ng/ml	Median (IQR)	7.0 (4.0–9.4)	114.5 (31.0–574.0)	** < 0.001**
3. AFP status (Abnormal/Normal)	n (%)/n (%)	0.0 (0.0)/125 (100.0)	83 (76.9)/25 (23.1)	** < 0.001**
IV. Hematological parameters				
1. WBCs, × 10^9^/L	Median (IQR)	7.8 (5.8–9.4)	5.3 (3.9–8.4)	** < 0.001**
2. RBCs, × 10^12^/L	Median (IQR)	4.5 (4.1–5.0)	3.7 (3.1–4.3)	** < 0.001**
3. Hematocrit (HCT), %	Median (IQR)	37.4 (34.1–41.6)	34.8 (30.2–39.6)	** < 0.001**
4. Hemoglobin, g/dl	Median (IQR)	13.5 (12.8–14.9)	11.9 (10.5–13.5)	** < 0.001**
5. Platelets count, × 10^9^/L	Median (IQR)	258.0 (198.0–338.0)	126.0 (93.5–163.0)	** < 0.001**

Data are presented as numbers with percentages or median with interquartile range. Fisher’s exact and two-sample Wilcoxon rank-sum tests were applied. Bold value indicates the *p*-value < 0.05.

IQR: interquartile range; HCC: hepatocellular carcinoma; ALT: Alanine transaminase; AST: aspartate transaminase; INR: International normalized ratio; Anti-HCV: hepatitis C virus autoantibodies; AFP: alpha-fetoprotein; WBCs: white blood cells; RBCs: red blood cells.

### The genotypic and allelic frequencies of the TERT variants

**Figure 2:**
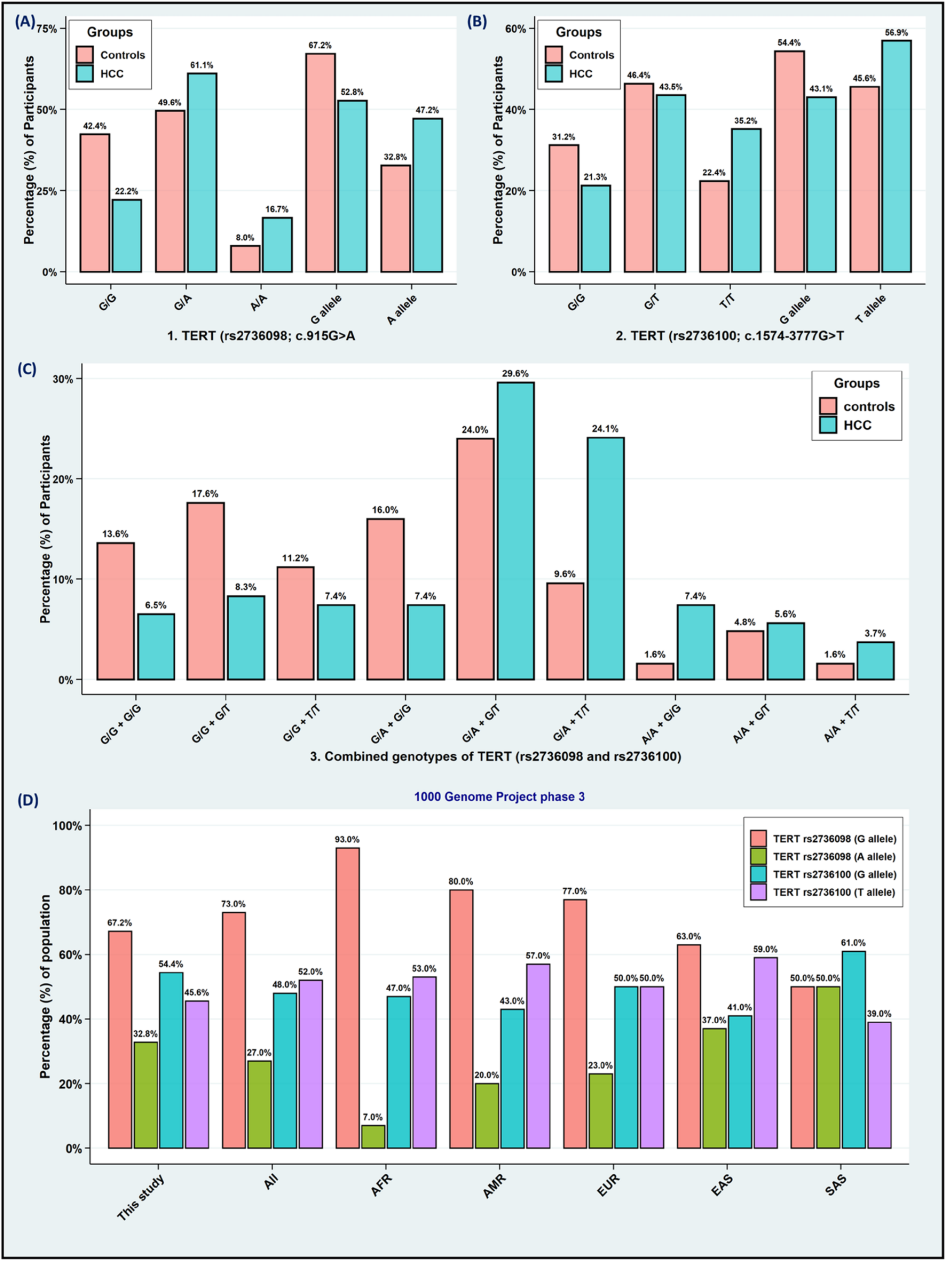
Allelic and genotypic distributions of the study population. (**A**): The allelic and genotypic frequencies of the *TERT (rs2736098; c.915G* > *A)* variant among HCC patients compared with cancer-free controls. (**B**): The allelic and genotypic frequencies of the *TERT (rs2736100; c.1574-3777G* > *T)* variant among HCC patients compared with cancer-free controls. (**C**): The combined genotypic frequencies of The *TERT (rs2736098 and rs2736100)* variants among HCC patients compared with cancer-free controls. (**D**) The 1000 Genome Project Phase 3 of the allelic frequencies for the present study compared with various ethnic populations (https://www.internationalgenome.org/). Abbreviations: AFR, Africa; AMR, America; EUR, Europe; EAS, East Asia; SAS, South Asia; HCC, Hepatocellular carcinoma.

The expected and observed frequencies of the TERT*(rs2736100; c.1574-3777G > *T) variant* for cancer-free controls and HCC patients were in alignment with the Hardy–Weinberg equilibrium (HWE) [*p*-value > 0.05], while those of the TERT*(rs2736098; c.915G > *A)* variant were mismatched with HWE among HCC patients [*p*-value = 0.02], and this could be imputed to the higher frequency of the TERT (rs2736098*G/A) genotype. Interestingly, the mutant genotypes of the TERT (rs2736098*A/A and rs2736100*T/T) were considerably elevated among HCC patients compared with control subjects [TERT (rs2736098*A/A): 16.7% vs. 8.0%, OR = 3.97, 95% CI = 1.60–9.89, *p*-value = 0.003; and TERT (rs2736100*T/T): 35.2% vs. 22.4%, OR = 2.30, 95% CI = 1.13–4.68, *p*-value = 0.023, respectively], (Table 2). Furthermore, the minor alleles of the TERT (rs2736098*A allele and rs2736100*T allele) were substantially associated with elevated risk of HCC compared with cancer-free controls [TERT (rs2736098*A allele): 47.2% vs. 32.8%, OR = 1.83, 95% CI = 1.26–2.67, *p*-value = 0.002; and TERT (rs2736100*T allele): 56.9% vs. 45.6%, OR = 1.58, 95% CI = 1.09–2.28, *p*-value = 0.016, respectively], (Fig. 2A & 2B). Besides that, the combined genotypes of TERT (rs2736098*G/A + rs2736100*T/T) and TERT (rs2736098*A/A + rs2736100*G/G) were statistically significant with an elevated risk of HCC compared to cancer-free controls [*p*-value = 0.004 and 0.047, respectively], (Fig. 2C). Upon establishing haplotype analysis of TERT*(rs2736098 and rs2736100) variants, our team identified a perceptible association of TERT (rs2736098*A and rs2736100*T) haplotypes among HCC patients compared with cancer-free controls [24.5% vs. 14.8%, OR = 1.87, 95% CI = 1.17–2.98, *p*-value = 0.009], (Table 2). Surprisingly, the minor allele frequency (MAF) among cancer-free controls for the TERT (rs2736098*A allele) was 0.33, while the minor allele frequency for the TERT (rs2736100*T allele) was 0.46. Based on the final phase of the 1000 genome project, the minor allele frequency of TERT (rs2736098*A allele) was matched with similar observations among East Asian (0.37) subjects, while those of TERT (rs2736100*T allele) was correlated with European (0.50) subjects, (Fig. 2D).

**Table 2:** Genotypic, allelic, and haplotypic frequencies of the *TERT (rs2736098 and rs2736100)* variants.

Genetic polymorphisms		Cancer-free controls	HCC patients	*OR (95% CI)*	*P*-value
1. TERT (rs2736098; c.915G > A)					
Genotypic frequencies		n (%) 125	n (%) 108		
G/G		53 (42.4)	24 (22.2)	1.0	
G/A		62 (49.6)	66 (61.1)	**2.35 (1.30–4.26)**	**0.006**
A/A		10 (8.0)	18 (16.7)	**3.97 (1.60–9.89)**	**0.003**
HWE		*χ*^2^ = *1.96, p* = *0.16*	*χ*^2^ = *5.52, ****p* = *0.02***		
Allelic frequencies		n (%) 250	n (%) 216		
G allele		168 (67.2)	114 (52.8)	1.0	
A allele		82 (32.8)	102 (47.2)	**1.83 (1.26–2.67)**	**0.002**
2. TERT (rs2736100; c.1574-3777G > T)					
Genotypic frequencies		n (%) 125	n (%) 108		
G/G		39 (31.2)	23 (21.3)	1.0	
G/T		58 (46.4)	47 (43.5)	1.37 (0.72–2.61)	0.417
T/T		28 (22.4)	38 (35.2)	**2.30 (1.13–4.68)**	**0.023**
HWE		*χ*^2^ = *0.52, p* = *0.47*	*χ*^2^ = *1.37, p* = *0.24*		
Allelic frequencies		n (%) 250	n (%) 216		
G allele		136 (54.4)	93 (43.1)	1.0	
T allele		114 (45.6)	123 (56.9)	**1.58 (1.09–2.28)**	**0.016**
3. Haplotypes					
rs2736098	rs2736100	n (%) 250	n (%) 216	*OR (95% CI)*	*P*-value
(G)	(G)	91 (36.4)	44 (20.4)	1.0	
(A)	(G)	45 (18.0)	49 (22.7)	1.34 (0.85–2.10)	0.247
(G)	(T)	77 (30.8)	70 (32.4)	1.08 (0.73–1.59)	0.764
(A)	(T)	37 (14.8)	53 (24.5)	**1.87 (1.17–2.98)**	**0.009**

Data are presented as numbers with percentages. Fisher’s exact test was applied.

OR, Odds Ratio; CI, Confidence Intervals; HWE, Hardy–Weinberg equilibrium. Bold values indicate *P*-value < 0.05.

Significant values are in [bold].

### Association of TERT variants with the susceptibility for HCC

Essentially, the distributions of the TERT*(rs2736098; c.915G > *A)* variant were significantly associated with elevated risk of HCC compared with control subjects under dominant [OR = 2.54, 95% CI = 1.43–4.53, *P*-value = 0.001], recessive [OR = 2.31, 95% CI = 1.01–5.27, *P*-value = 0.042], and homozygote [OR = 3.95, 95% CI = 1.58–9.85, *P*-value = 0.002] models. Moreover, the frequencies of the TERT*(rs2736100; c.1574-3777G > *T)* variant revealed a statistical difference with increased risk of HCC compared to control subjects under recessive [OR = 1.84, 95% CI = 1.03–3.29, *P*-value = 0.039] model, (Table 3).

**Table 3:** Genetic association models of the *TERT (rs2736098 and rs2736100)* variants with the risk of hepatocellular carcinoma.

Model	Genotypes	Cancer-free controls	HCC patients	Crude OR (95% CI)	*P*-value	Adjusted OR (95% CI)	*P*-value
*1. TERT (rs2736098; c.915G* > *A)*		n (%) 125	n (%) 108				
Codominant	G/G	53 (42.4)	24 (22.2)	1.0	**0.002**	1.0	**0.002**
	G/A	62 (49.6)	66 (61.1)	**2.35 (1.30–4.26)**		**2.31 (1.27–4.20)**	
	A/A	10 (8.0)	18 (16.7)	**3.97 (1.60–9.89)**		**3.95 (1.58–9.85)**	
Dominant	G/G	53 (42.4)	24 (22.2)	1.0	**0.001**	1.0	**0.001**
	G/A + A/A	72 (57.6)	84 (77.8)	**2.58 (1.45–4.58)**		**2.54 (1.43–4.53)**	
Recessive	G/G + G/A	115 (92.0)	90 (83.3)	1.0	**0.042**	1.0	**0.042**
	A/A	10 (8.0)	18 (16.7)	**2.30 (1.01–5.23)**		**2.31 (1.01–5.27)**	
Overdominant	G/G + A/A	63 (50.4)	42 (38.9)	1.0	0.078	1.0	0.089
	G/A	62 (49.6)	66 (61.1)	1.60 (0.95–2.69)		1.57 (0.93–2.66)	
Log-additive	–	–	–	**2.09 (1.36–3.21)**	** < 0.001**	**2.08 (1.35–3.19)**	** < 0.001**
*2. TERT (rs2736100; c.1574-3777G* > *T)*		n (%) 125	n (%) 108				
Codominant	G/G	39 (31.2)	23 (21.3)	1.0	0.060	1.0	0.069
	G/T	58 (46.4)	47 (43.5)	1.37 (0.72–2.61)		1.41 (0.74–2.70)	
	T/T	28 (22.4)	38 (35.2)	2.30 (1.13–4.68)		2.29 (1.12–4.69)	
Dominant	G/G	39 (31.2)	23 (21.3)	1.0	0.086	1.0	0.081
	G/T + T/T	86 (68.8)	85 (78.7)	1.68 (0.92–3.04)		1.70 (0.93–3.10)	
Recessive	G/G + G/T	97 (77.6)	70 (64.8)	1.0	**0.031**	1.0	**0.039**
	T/T	28 (22.4)	38 (35.2)	**1.88 (1.06–3.35)**		**1.84 (1.03–3.29)**	
Overdominant	G/G + T/T	67 (53.6)	61 (56.5)	1.0	0.660	1.0	0.750
	G/T	58 (46.4)	47 (43.5)	0.89 (0.53–1.49)		0.92 (0.54–1.55)	
Log-additive	–	–	–	**1.52 (1.07–2.17)**	**0.019**	**1.52 (1.06–2.17)**	**0.022**

Data are presented as numbers with percentages. Chi squared test was applied. Adjusted by age and gender.

OR, Odds Ratio; CI, Confidence Intervals. Bold values indicate *P*-value < 0.05.

### Association of TERT variants with the clinical and laboratory variables

**Figure 3:**
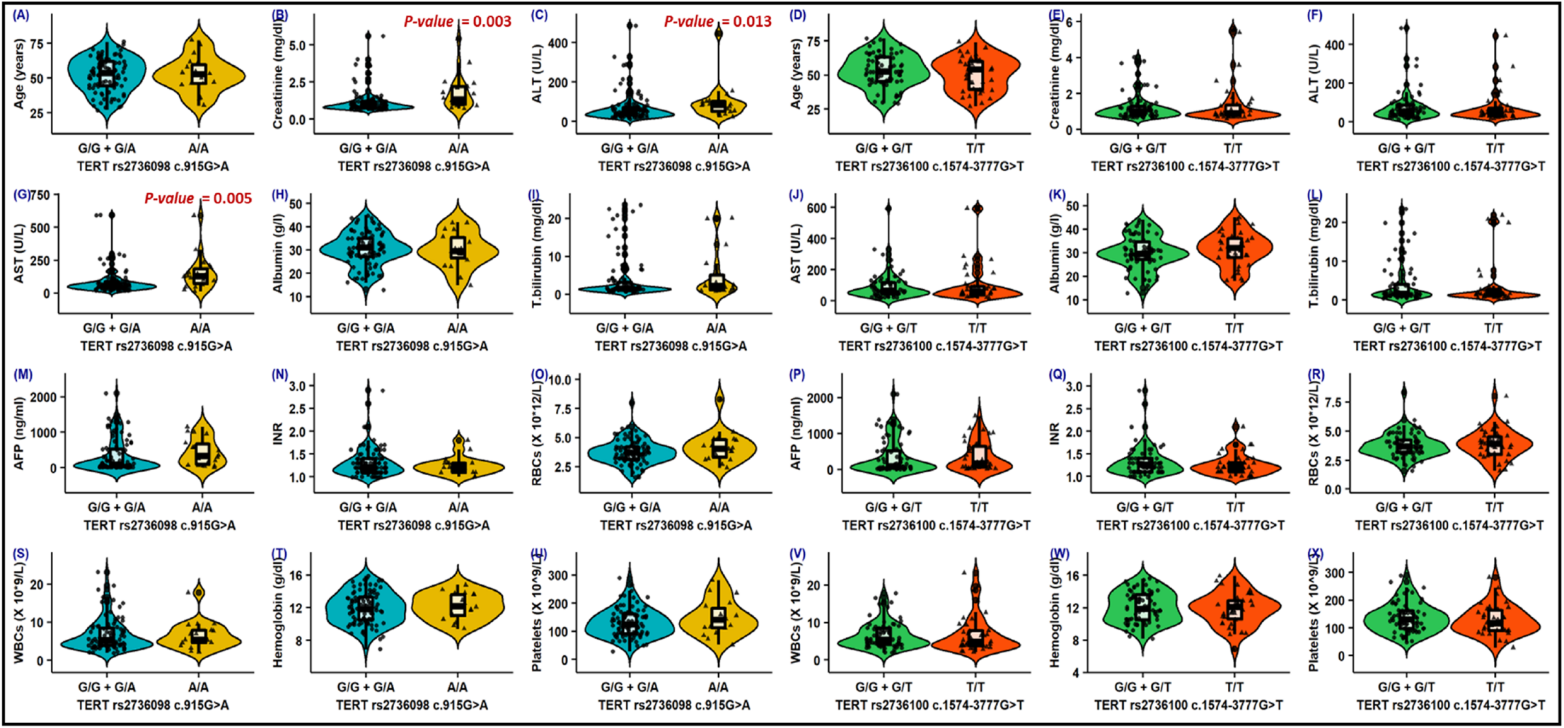
Impact of *TERT* variants on the clinical, serological, and laboratory parameters. Violin plot combined with box plot for the distribution of *TERT (rs2736098 and rs2736100)* variants with the clinical, serological, laboratory, and hematological parameters.

Remarkably, the TERT*(rs2736098; c.915G > *A)* variant was significantly correlated with higher levels of ALT, AST, and creatinine, (Fig. 3). Additionally, the TERT (rs2736098*A/A) genotype revealed a noticeable association with positive smoking and splenomegaly (*p*-value < 0.05). Alternatively, the TERT (rs2736100*T/T) genotype identified a significant association with elevated levels of HCV autoantibodies (*p*-value = 0.009), (Table S1).

### Multivariate analysis of TERT variants

**Figure 4:**
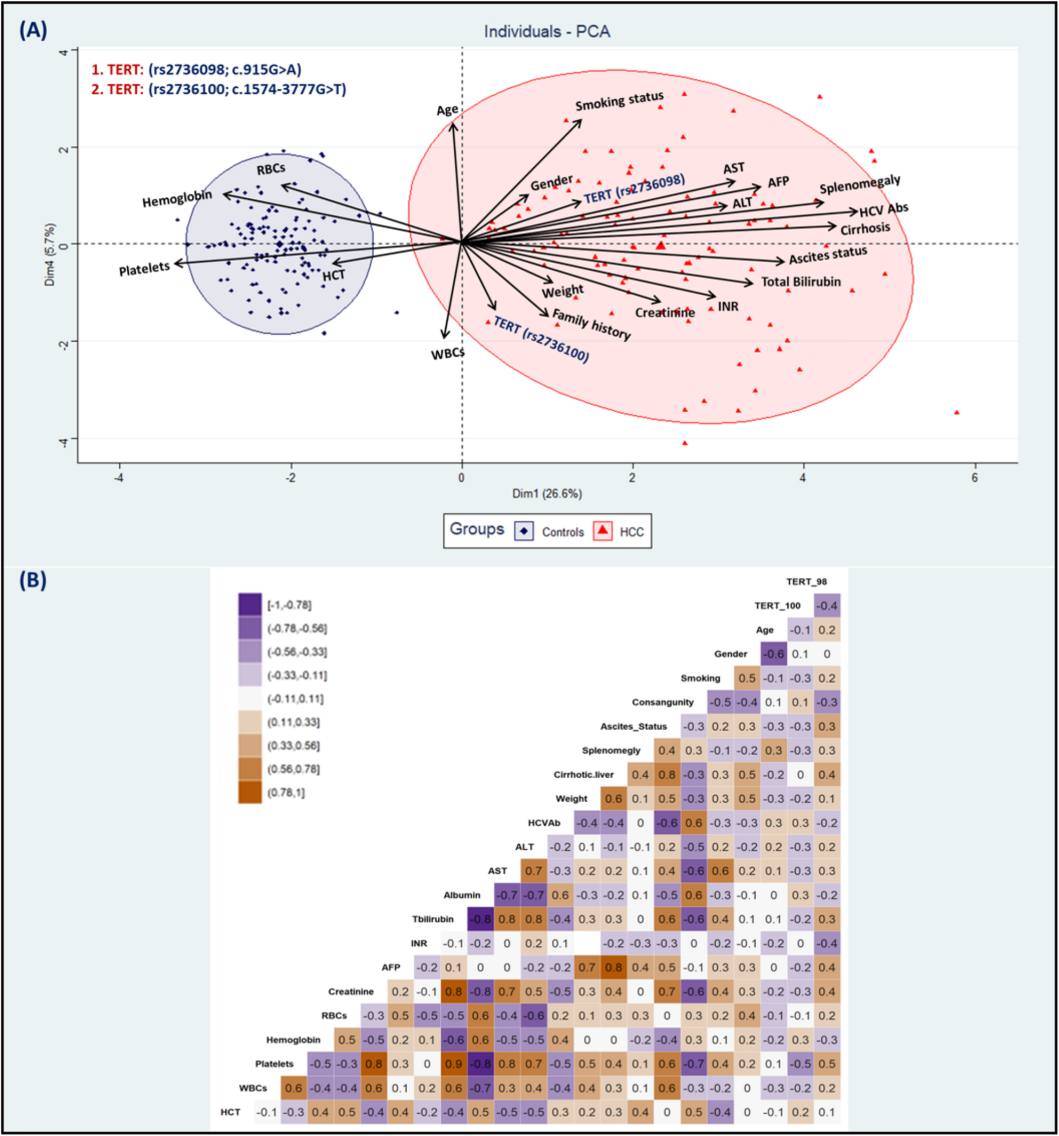
Multivariate analysis of the *TERT (rs2736098 and rs2736100)* variants. (**A**): Principal component analysis (PCA) of the study participants revealed a definite distinction between HCC patients and cancer-free controls. Elongated arrows represented elevated impact of clinical, serological, and laboratory investigations among the study participants. Visually, the *TERT (rs2736098; c.915G* > *A)* and *TERT (rs2736100; c.1574-3777G* > *T)* variants exhibited a significant association with elevated risk of hepatocellular carcinoma. (**B**): Correlation matrix using Pearson’s correlation test of the clinical, serological measurements, and laboratory variables among HCC patients.

Firstly, our team performed the principal component analysis (PCA) that clustered the study participants into two categories involving cancer-free controls and HCC patients, (Fig. 4A). The PCA technique revealed an obvious distinction between the two groups with elongated arrows exhibiting a significant impact on the clinical, serological, laboratory, and hematological variables. By observation, the TERT*(rs2736098; c.915G > *A)* and TERT*(rs2736100; c.1574-3777G > *T)* variants exhibited a significant difference with an elevated risk of hepatocellular carcinoma. On the other hand, the correlation matrix was carried out utilizing Pearson’s correlation test to assess the conventional possibilities among HCC patients for the clinical characteristics, and laboratory investigations, (Fig. 4B).

### In silico data analysis using computational bioinformatics tools

**Figure 5:**
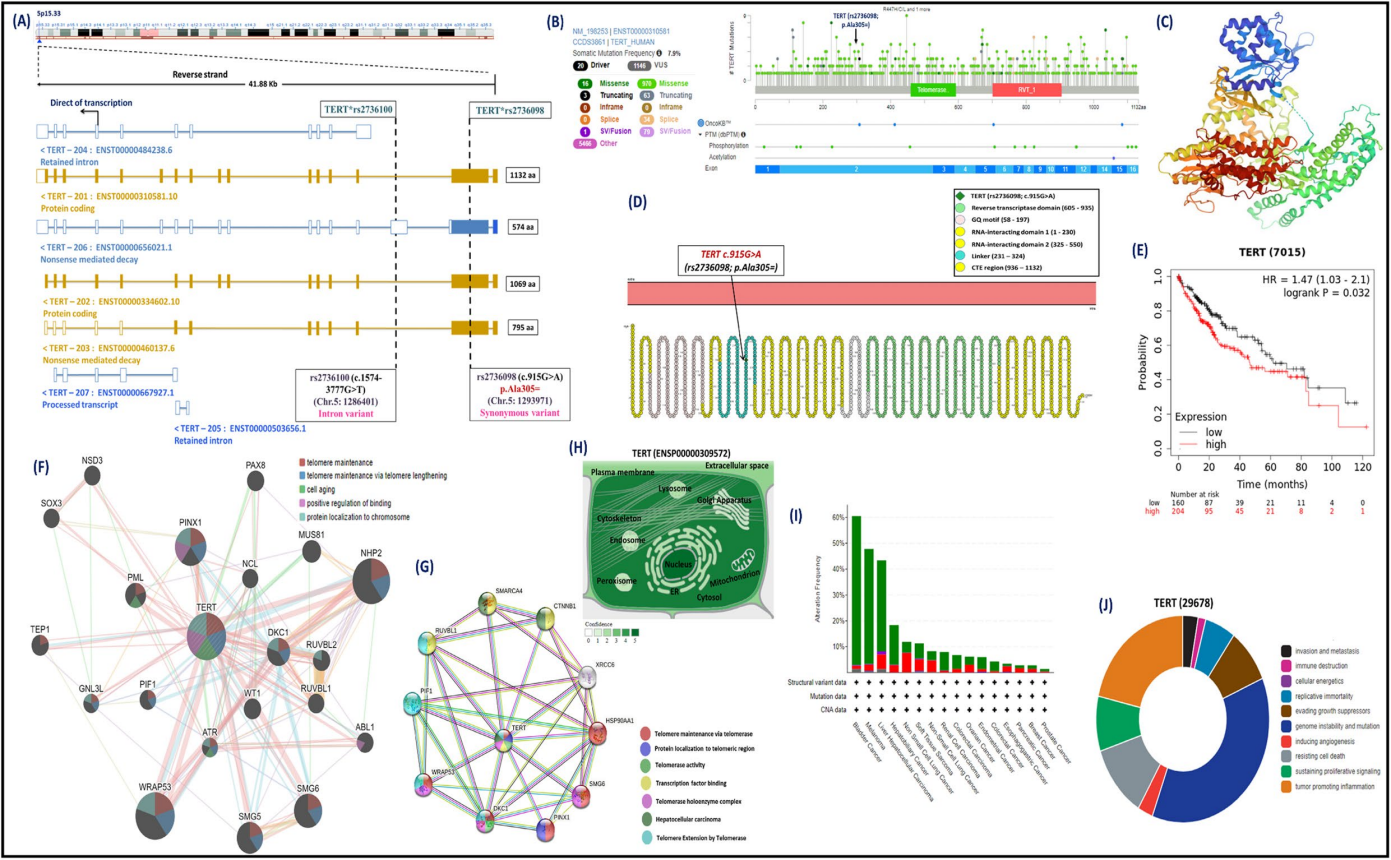
The computational bioinformatic tools of the *TERT* gene. (**A**): The chromosomal localization and genomic structure of the *TERT* gene. It is positioned on the chromosome number 5p15.33 and spanned about 41,922 bases [Chr.5:1253147–1295068] that is orientated along the minus strand. The genomic structure of *TERT* gene identified that is comprised of seven splice transcripts with the main one (TERT-201) including 16 exons and 15 introns. (**B**): Lollipop diagram of the *TERT gene* mutations derived from several databases for studies indicating the presence of different somatic mutation with a frequency of 7.9% and observing the *TERT (rs2736098; p.Ala305* =*)* variant at the second exon of the gene. **(C**): The crystalline structure of human TERT protein using Protein Data Bank. (**D**): Amino acid residues of the TERT protein (O14746-1) and its domains showing the locations of the *TERT (rs2736098; c.915G* > *A; p.Ala305* =*)*. (**E**): Survival analysis during high and low TERT expression. (**F**): Gene–gene interactions of the *TERT gene* using GeneMANIA database. (**G**): Protein–protein interactions of the TERT protein using the STRING database. (**H**): Subcellular localization of the TERT protein, with darker colors suggesting further abundance. (**I**): Mutual exclusive analysis of the TERT expression in different cancerous diseases. (**J**): TERT is involved in the development of cancer hallmarks. [Data source: Ensembl.org, NCBI database, UniProt database, Compartment database, Protter database, Kaplan‐Meier plotter database, GeneMania, STRING version 11.0, cBioPortal cancer Genomics database, Cancer Hallmarks Analytics tool, and Protein Data Bank].

The chromosomal localization of the *TERT gene* (ENSG00000164362) is positioned on 5p15.33 and spanned about 41,922 bases [Chr.5: 1253147–1295068] that is orientated along the minus strand. The genomic localization of the *TERT* gene comprised of seven splice variants with the main transcript (TERT-201) including 16 exons and 15 introns, with 1132 amino acids. Besides that, the TERT*(rs2736098; c.915G > *A)* variant is located on the second exon (Chr.5: 1293971), while the TERT*(rs2736100; c.1574-3777G > *T)* variant is situated on the second intron (Chr.5: 1286401) (Fig. 5). The somatic mutation frequency of the *TERT* gene that was extracted from several databases was 7.9%, indicating the presence of the TERT*(rs2736098; p.Ala305 =*)* variant at the second exon of the gene. The crystalline structure and amino acid residues of the TERT protein (O14746-1) and its domains representing the location of the *TERT (rs2736098; p.Ala305* =*)* [Data source: Protter database, UniProt database, and Protein Data Bank]. Moreover, the Kaplan–Meier plotter database of hepatic cancer indicates elevated TERT expression with a worse prognosis (*p*-value = 0.032). The gene–gene associations of the *TERT gene* revealed the contribution of this gene in telomere maintenance, telomere lengthening, cell aging, and protein localization to chromosomes. The protein–protein frameworks identified the potential role of the TERT protein in telomere maintenance, protein localization to telomeric region, telomerase activity, transcription factor binding, and telomere extension by telomerase. The subcellular localizations of the TERT protein suggest high levels of profusion in the nucleus, cytosol, mitochondrion, and cytoskeleton. Additionally, mutual exclusive analysis of the TERT expression within different cancerous diseases implicated the higher rate of liver hepatocellular carcinoma. Lastly, our team identified the crucial role of the *TERT* gene in the development of cancer hallmarks.

## Discussion

Recently, three crucial factors were thought to control the telomere lengthening involving telomerase activity, high mitotic rate, and telomerase-dependent variables^6,40^. However, the ability of the *TERT* gene to encode a regulatable enzyme that controls the telomerase expression and telomere lengthening through inserting repetitive nucleotide units (5′-TTAGGG-3′) at the chromosomal terminus could determine the proliferation level of carcinoma ^18,24^. Upon thorough literature searching for the reports identifying the association of the TERT*(rs2736098 and rs2736100) variants with the progression of hepatocellular carcinoma, our team confirmed that this work is the innovative one for testing the correlation of the TERT*(rs2736100; c.1574-3777G > *T)* variant with elevated risk of HCC among Egyptian subjects.

Remarkably, our findings revealed a significant difference for the TERT*(rs2736098; c.915G > *A)* variant and elevated risk of HCC under allelic, recessive, and dominant models (OR = 1.83, 2.31, and 2.54, respectively). Obviously, HCC patients carrying TERT (rs2736098*AA) genotype exposed a statistically significant for positive smoking, and levels of AST/ALT compared to control subjects (*p*-value < 0.05). On the other hand, the TERT*(rs2736100; c.1574-3777G > *T)* variant was identified significantly associated with elevated risk of HCC under allelic (rs2736100*T), and recessive (rs2736100*TT) models (OR = 1.58, and 1.84, respectively). Additionally, HCC patients carrying the TERT (rs2736100*TT) genotype conferred a significant difference with higher levels of autoantibodies for anti-HCV compared to control subjects (*p*-value = 0.009). Interestingly, the higher levels of ALT, AST, and HCV autoantibodies with HCC development were confirmed and studied in previous reports^41–43^. Several meta-analysis reports investigated the association of the *TERT* gene variants with the susceptibility for hepatocellular carcinoma, but with challenging interpretations^6,18,44^. A recent meta-analysis study indicated the contribution of the TERT*(rs2736098; c.915G > *A)* variant with the susceptibility for HCC among overall subjects under the dominant model (GA + AA vs. GG; OR = 1.38)^44^. On the contrary, a meta-analysis report revealed a strong association between the TERT*(rs2736098 and rs2736100) variants with increased risk of different types of carcinomas among overall subjects^6^. However, they failed to indicate a significant correlation between the TERT*(rs2736098; c.915G > *A)* and TERT*(rs2736100; c.1574-3777G > *T)* variants and the HCC progression among Asian and Caucasian subjects^6^. Another meta-analysis study confirmed no evidence for the association between TERT*(rs2736100; c.1574-3777G > *T)* variant and the susceptibility of different digestive cancers under various genetic models within Asian and Caucasian subjects^18^.

Owing the inconsistency of findings, our team was motivated to construct a systematic comparison for all the articles investigated the correlation of the TERT*(rs2736098 and rs2736100) variants with the HCC susceptibility among different ethnic groups (Table 4). Interestingly, two comparisons identified a significant association between the TERT*(rs2736098; c.915G > *A)* variant and elevated risk of HCC among Chinese subjects (OR = 1.88 and 1.59, respectively)^45,46^. On the other hand, three reports confirmed no significant difference for the TERT*(rs2736098; c.915G > *A)* variant and increased risk of HCC among Chinese subjects^26,28^, and Egyptian subjects^27^ under different genetic models. Limited data is available concerning the contribution of the TERT*(rs2736100; c.1574-3777G > *T)* variant with the progression of HCC among various ethnic groups. A single report done among Chinese subjects revealed a statistically significant for the TERT*(rs2736100; c.1574-3777G > *T)* variant with increased risk of HCC under dominant model (OR = 1.61, *p*-value = 0.047)^26^. Conversely, another study among Chinese subjects observed no indication of association for the TERT*(rs2736100; c.1574-3777G > *T)* variant with the HCC progression under dominant and recessive models^28^. As exemplified in the forest plots shown in (Figure S3) for the pooled data concerning the association between TERT*(rs2736098 and rs2736100) variants and HCC progression, our team revealed a strong association for the TERT*(rs2736098; c.915G > *A)* variant with elevated risk of HCC under dominant model (OR = 1.37, *p*-value = 0.041).

**Table 4:** Worldwide distribution of the *TERT (rs2736098 and rs2736100)* variants among HCC patients compared with cancer-free controls.

References	Country	Ethnicity	HCC patients				Cancer-free controls				Dominant model		Recessive model	
*1. TERT (rs2736098; c.915G* > *A)*			N	GG	GA	AA	N	GG	GA	AA	OR (95% CI)	*P*-value	OR (95% CI)	*P*-value
This study [2023]	Egypt	African	108	24	66	18	125	53	62	10	**2.58 (1.45–4.58)**	**0.001**	**2.30 (1.01–5.23)**	**0.047**
Sharaf^27^	Egypt	African	70	37	28	5	40	18	17	5	0.73 (0.33–1.59)	0.428	0.54 (0.15–1.99)	0.353
Yuan^26^	China	Asian	231	85	127	19	240	94	115	31	1.11 (0.76–1.60)	0.596	0.60 (0.33–1.10)	0.101
Su^45^	China	Asian	201	75	97	29	210	111	76	23	**1.88 (1.27–2.79)**	**0.002**	1.37 (0.76–2.46)	0.291
Zhang^46^	China	Asian	400	133	206	61	400	177	158	65	**1.59 (1.20–2.12)**	**0.001**	0.93 (0.63–1.36)	0.698
Ding^28^	China	Asian	1273	500	563	210	1328	526	604	198	1.01 (0.87–1.19)	0.863	1.13 (0.91–1.39)	0.266

*2. TERT (rs2736100; c.1574-3777G* > *T)*			N	GG	GT	TT	N	GG	GT	TT	OR (95% CI)	*P*-value	OR (95% CI)	*P*-value
This study [2023]	Egypt	African	108	23	47	38	125	39	58	28	1.68 (0.92–3.04)	0.090	**1.88 (1.06–3.35)**	**0.032**
Yuan^26^	China	Asian	201	35	92	74	237	60	108	69	**1.61 (1.01–2.57)**	**0.047**	1.42 (0.95–2.12)	0.087
Ding^28^	China	Asian	1269	208	633	428	1322	222	651	449	1.03 (0.84–1.27)	0.783	0.99 (0.84–1.16)	0.899

Data are presented as numbers.

OR, Odds Ratio; CI, Confidence Intervals. Bold values indicate *P*-value < 0.05.

Upon precise searching for online electronic databases that could predict the potential impact of the intronic variant of the TERT*(rs2736100; c.1574-3777G > *T)* gene using RegulomeDB annotation for regulatory elements in non-coding regions, we could predict that this variant has a probability score equal to 0.13^47^. Another predicted tool for exploring annotations of the noncoding variants is HaploReg that extracted their information from linkage disequilibrium (LD) of the final phase of 1000 genome project ^48^. Interestingly, the TERT*(rs2736100; c.1574-3777G > *T)* variant revealed linkage disequilibrium with (*r*^2^ ≥ 0.8) and could induce the expression of transcriptional factor within the Foxa subfamily of Forkhead box protein A (Foxa) factors^49^. These Foxa factors also are called as hepatocyte nuclear factor 3 (HNF3) that have a potential function in hepatic specification through controlling in the hepatic organogenesis and repositioning chromatin and nucleosomes^49,50^. From the above-mentioned outcomes, we could consider that the TERT (rs2736098*A allele) and TERT (rs2736100*T allele) variants were correlated with elevated risk of HCC and telomere shortening in the late onset of the disease. Lastly, minor limitations were recognized during the establishment of this research including the relatively small sample size, formulating this work among Egyptian subjects, the case–control design, the lack of functional analysis, and the absence of accounting for potential confounding factors. Replication and validation of the findings in larger and more diverse cohorts, along with functional studies and control of confounding variables, would further strengthen the upcoming studies. Overall, this study contributes to the understanding of the potential role of TERT gene variants in HCC susceptibility among Egyptian subjects, highlighting the need for further research in this area.

## Conclusion

This work indicated the potential role of the TERT (rs2736098*A and rs2736100*T) alleles with elevated risk of HCC among Egyptian patients. Interestingly, the TERT (rs2736098*A) and TERT (rs2736100*T) alleles were correlated with telomere shortening in the late onset of the disease. Thus, the TERT*(rs2736098; c.915G > *A)* and TERT*(rs2736100; c.1574-3777G > *T)* variants could be thought as independent risk factors with the progression of HCC.

### Supplementary Information

Supplementary Information 1.

Supplementary Information 2.

## Data Availability

The data that support the findings of this study are available from the corresponding author upon reasonable request.
