# Supplementary figures and images for: Association of TERT (rs2736098 and rs2736100) genetic variants with elevated risk of hepatocellular carcinoma: a retrospective case–control study
Source: Sci Rep. 2023 Oct 26;13:18382. doi: 10.1038/s41598-023-45716-w (PMC10603040; doi:10.1038/s41598-023-45716-w)

**Supplementary file (1)**

**The original gel images and cropped ones:**

1. **TERT*(rs2736098)**


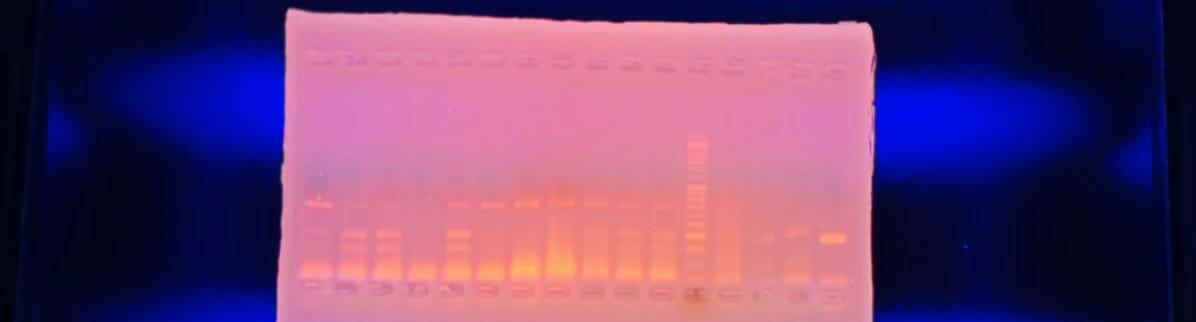


***
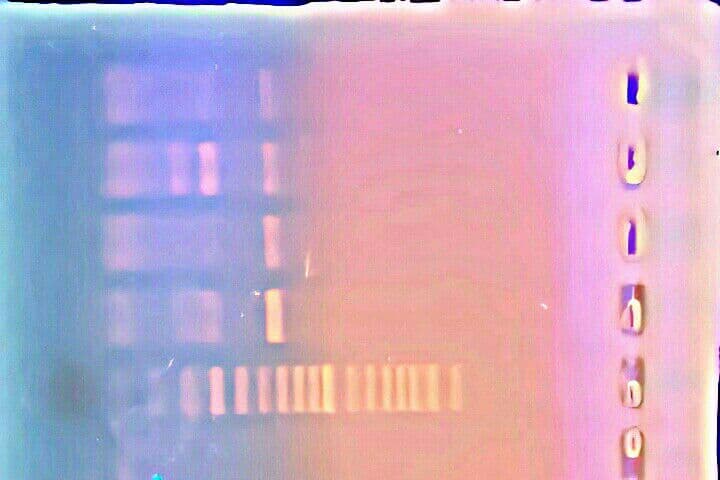
***


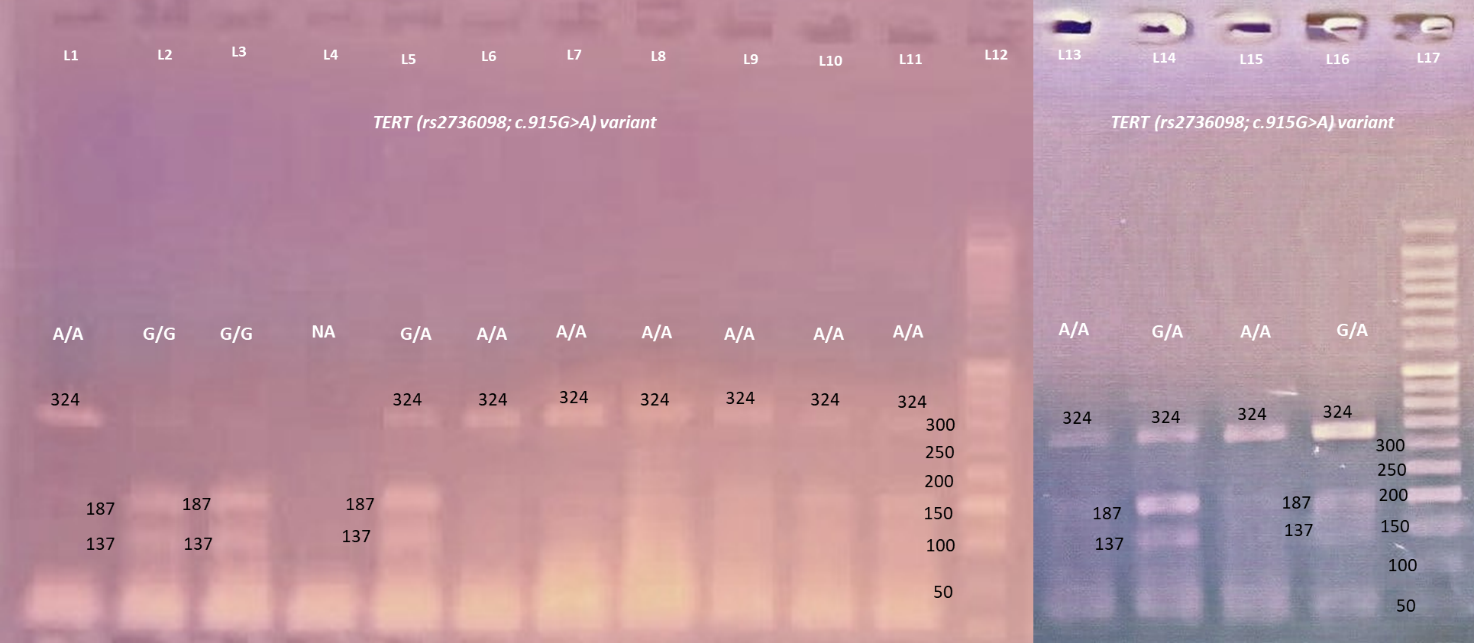


1. **TERT*(rs2736100)**


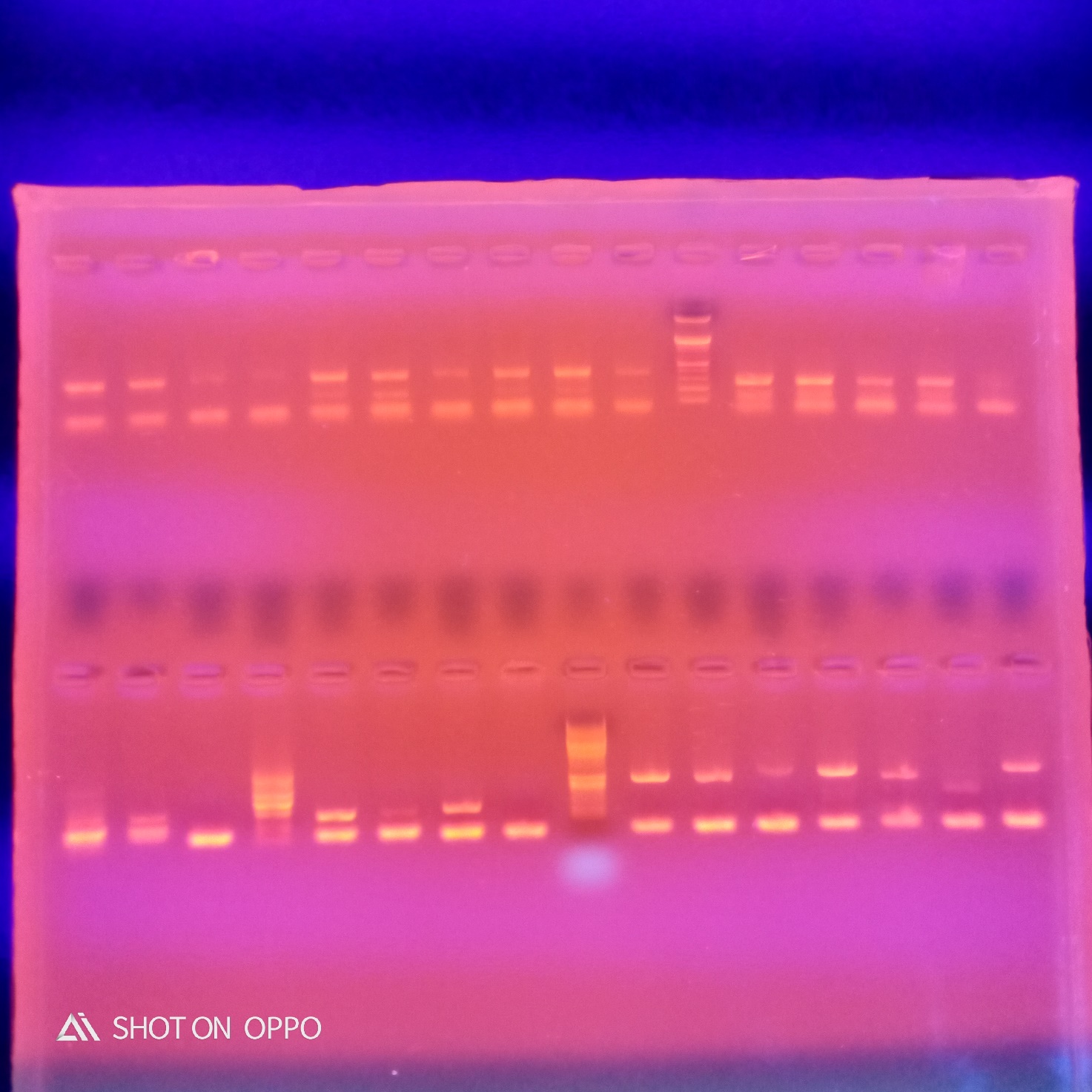


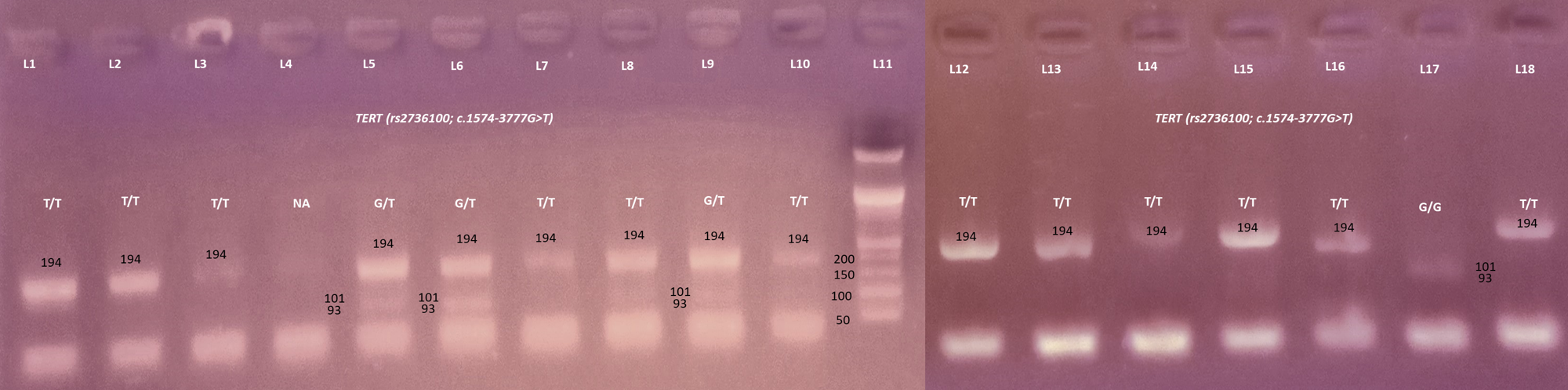

Supplement: Supplementary file 1 — Supplementary Information 1. [file 41598_2023_45716_MOESM1_ESM.docx]
