# Supplementary material for: Association of TERT (rs2736098 and rs2736100) genetic variants with elevated risk of hepatocellular carcinoma: a retrospective case–control study
Source: Sci Rep. 2023 Oct 26;13:18382. doi: 10.1038/s41598-023-45716-w (PMC10603040; doi:10.1038/s41598-023-45716-w)
Supplement: Supplementary file 2 — Supplementary Information 2. [file 41598_2023_45716_MOESM2_ESM.docx]

****Association of *TERT (rs2736098 and rs2736100)* genetic variants with elevated risk of hepatocellular carcinoma: a retrospective case-control study****

**Table S1. Genotypic frequencies of *TERT (rs2736098 and rs2736100)* variants stratified by the demographic, clinical, and laboratory variables among HCC patients.**

| **Parameters** | | ***TERT (rs2736098; c.915G>A)*** | | | ***TERT (rs2736100; c.1574-3777G>T)*** | | |
| --- | --- | --- | --- | --- | --- | --- | --- |
|  |  | **A/A** | **G/G + G/A** | ***p-value*** | **T/T** | **G/G + G/T** | ***p-value*** |
|  |  | **(n= 18)** | **(n= 90)** |  | **(n= 38)** | **(n= 70)** |  |
| ***I. Demographic and clinical characteristics*** | | | | | | | |
| ***1. Age, years*** | *Median (IQR)* | 52.5 (46.0-60.0) | 53.5 (44.0-62.0) | 0.997 | 54.0 (39.0-60.0) | 52.5 (45.0-63.0) | 0.383 |
| ***2. Age groups, years (≤40/>40)*** | *n (%)/n (%)* | 3 (16.7)/15 (83.3) | 18 (20.0)/72 (80.0) | 0.744 | 11 (28.9)/27 (71.1) | 10 (14.3)/60 (85.7) | 0.066 |
| ***3. Weight, kg*** | *Median (IQR)* | 82.5 (74.0-89.0) | 85.0 (78.0-89.0) | 0.650 | 85.0 (80.0-90.0) | 82.5 (77.0-89.0) | 0.330 |
| ***4. Gender (Male/Female)*** | *n (%)/n (%)* | 14 (77.8)/4 (22.2) | 74 (82.2)/16 (17.8) | 0.658 | 32 (84.2)/6 (15.8) | 56 (80.0)/14 (20.0) | 0.591 |
| ***5. Smoking (Positive/Negative)*** | *n (%)/n (%)* | 10 (55.6)/8 (44.4) | 22 (24.4)/68 (75.6) | **0.008** | 11 (28.9)/27 (71.1) | 21 (30.0)/49 (70.0) | 0.909 |
| ***6. Consanguinity (Positive/Negative)*** | *n (%)/n (%)* | 3 (16.7)/15 (83.3) | 23 (25.6)/67 (74.4) | 0.421 | 9 (23.7)/29 (76.3) | 17 (24.3)/53 (75.7) | 0.944 |
| ***7. Family history (Positive/Negative)*** | *n (%)/n (%)* | 3 (16.7)/15 (83.3) | 16 (17.8)/74 (82.2) | 0.910 | 7 (18.4)/31 (81.6) | 12 (17.1)/58 (82.9) | 0.868 |
| ***8. Cirrhotic liver (Positive/Negative)*** | *n (%)/n (%)* | 14 (77.8)/4 (22.2) | 65 (72.2)/25 (27.8) | 0.627 | 29 (76.3)/9 (23.7) | 50 (71.4)/20 (28.6) | 0.584 |
| ***9. Hypertension (Positive/Negative)*** | *n (%)/n (%)* | 2 (11.1)/16 (88.9) | 31 (34.4)/59 (65.6) | 0.050 | 14 (36.8)/24 (63.2) | 19 (27.1)/51 (72.9) | 0.296 |
| ***10. Ascites status (Presence/Absence)*** | *n (%)/n (%)* | 14 (77.8)/4 (22.2) | 58 (64.4)/32 (35.6) | 0.273 | 24 (63.2)/14 (36.8) | 48 (68.6)/22 (31.4) | 0.569 |
| ***11. Splenomegaly (Presence/Absence)*** | *n (%)/n (%)* | 18 (100.0)/0 (0.0) | 76 (84.4)/14 (15.6) | **0.015** | 32 (84.2)/6 (15.8) | 62 (88.6)/8 (11.4) | 0.716 |
| ***II. Biochemical measurements*** | | | | | | | |
| ***1. ALT, U/L*** | *Median (IQR)* | 84.0 (49.0-102.0) | 48.0 (32.0-72.0) | **0.013** | 46.5 (32.0-70.0) | 55.0 (34.0-87.0) | 0.455 |
| ***2. AST, U/L*** | *Median (IQR)* | 128.5 (65.0-194.0) | 59.5 (40.0-88.0) | **0.005** | 57.0 (37.0-92.5) | 64.5 (45.0-116.0) | 0.719 |
| ***3. Albumin, g/l*** | *Median (IQR)* | 29.5 (26.0-36.0) | 31.0 (27.0-35.0) | 0.779 | 32.0 (28.0-36.0) | 29.0 (27.0-35.0) | 0.192 |
| ***4. Total bilirubin, mg/dl*** | *Median (IQR)* | 2.15 (1.10-5.60) | 1.50 (1.10-3.10) | 0.330 | 1.45 (1.10-2.70) | 1.60 (1.10-4.20) | 0.469 |
| ***5. Direct bilirubin, mg/dl*** | *Median (IQR)* | 1.04 (0.65-3.74) | 0.81 (0.42-2.01) | 0.194 | 0.86 (0.47-1.64) | 0.84 (0.50-2.45) | 0.690 |
| ***6. Indirect bilirubin, mg/dl*** | *Median (IQR)* | 0.83 (0.55-1.86) | 0.79 (0.56-1.15) | 0.609 | 0.71 (0.56-0.98) | 0.87 (0.55-1.58) | 0.339 |
| ***7. International normalized ratio (INR)*** | *Median (IQR)* | 1.20 (1.10-1.30) | 1.20 (1.10-1.40) | 0.486 | 1.20 (1.10-1.30) | 1.25 (1.10-1.40) | 0.266 |
| ***8. Creatinine, mg/dl*** | *Median (IQR)* | 1.32 (1.00-2.20) | 0.90 (0.80-1.20) | **0.003** | 0.90 (0.80-1.37) | 1.00 (0.80-1.30) | 0.276 |
| ***III. Serological investigations and Tumor markers*** | | | | | | | |
| ***1. Anti-HCV (positive/negative)*** | *n (%)/n (%)* | 15 (83.3)/3 (16.7) | 73 (81.1)/17 (18.9) | 0.825 | 36 (94.7)/2 (5.3) | 52 (74.3)/18 (25.7) | **0.009** |
| ***2. AFP, ng/ml*** | *Median (IQR)* | 325.0 (70.0-700.0) | 79.5 (27.0-521.0) | 0.083 | 165.0 (60.0-650.0) | 81.0 (28.0-521.0) | 0.419 |
| ***3. AFP status (abnormal/normal)*** | *n (%)/n (%)* | 17 (94.4)/1 (5.56) | 66 (73.3)/24 (26.7) | 0.053 | 32 (84.2)/6 (15.8) | 51 (72.9)/19 (27.1) | 0.182 |
| ***IV. Hematological parameters*** | | | | | | | |
| ***1. WBCs, x 10^9^/L*** | *Median (IQR)* | 5.35 (4.60-7.90) | 5.25 (3.90-8.50) | 0.786 | 4.85 (3.70-7.90) | 5.40 (4.10-8.50) | 0.571 |
| ***2. RBCs, x 10^12^/L*** | *Median (IQR)* | 4.05 (3.20-4.86) | 3.70 (3.10-4.20) | 0.197 | 3.90 (3.00-4.60) | 3.70 (3.20-4.28) | 0.569 |
| ***3. Hematocrit (HCT), %*** | *Median (IQR)* | 35.3 (32.9-39.9) | 34.7 (30.0-39.4) | 0.339 | 35.7 (30.4-39.0) | 34.7 (30.1-40.0) | 0.757 |
| ***4. Hemoglobin, g/dl*** | *Median (IQR)* | 12.1 (11.0-13.6) | 11.8 (10.5-13.3) | 0.656 | 12.1 (10.5-12.9) | 11.8 (10.5-13.6) | 0.690 |
| ***5. Platelet count, x 10^9^/L*** | *Median (IQR)* | 140.5 (112.0-190.0) | 124.0 (91.0-162.0) | 0.303 | 116.0 (90.0-165.0) | 130.5 (97.0-162.0) | 0.333 |
| ***Note*:** Data are presented as numbers with percentages or median with interquartile range. Chi square and two-sample Wilcoxon rank-sum tests were applied. Bold value indicates the p-value < 0.05.  ***Abbreviations*:** IQR: interquartile range; HCC: hepatocellular carcinoma; TERT: telomerase reverse transcriptase; ALT: Alanine transaminase; AST: aspartate transaminase; INR: International normalized ratio; Anti-HCV: hepatitis C virus antibodies; AFP: alpha-fetoprotein; WBCs: white blood cells; RBCs: red blood cells. | | | | | | | |


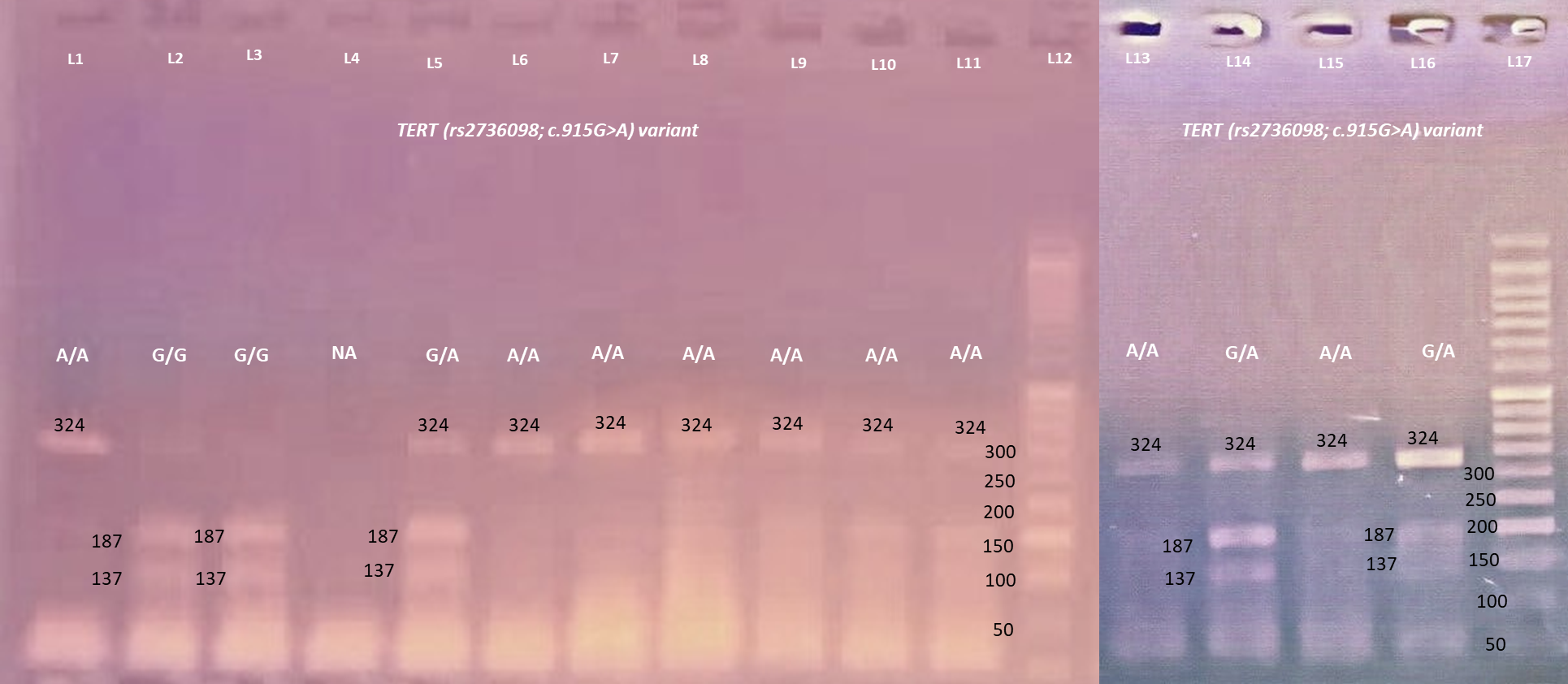


**Fig. S1. Photograph showing agarose gel electrophoresis (2.5%) of the processed fragments for the *TERT (rs2736098; c.915G>A)* *variant* using PCR-RFLP technique.** Lanes 1, 6, 7, 8, 9, 10, 11, 13, and 15 indicate mutant genotype*(A/A) at 324 bp. Lanes 2 and 3 represented homozygote genotype*(G/G) at 137 & 187 bp, while Lanes 5, 14, and 16 showed heterozygote genotype*(G/A) at 324, 137, and 187 bp. Lanes 12 and 17 identify DNA size marker (50‐1000Kbp).


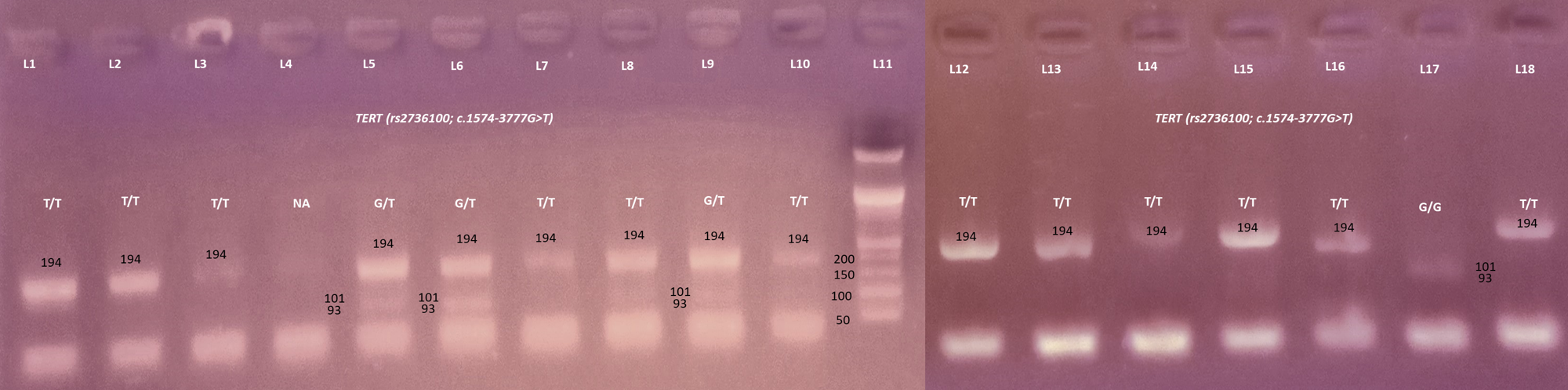


**Fig. S2. Photograph showing agarose gel electrophoresis (2.5%) of the processed fragments for the *TERT (rs2736100; c.1574-3777G>T) variant* using PCR-RFLP technique.** Lanes 1, 2, 3, 7, 8, 10, 12, 13, 14, 15, 16, and 18 indicate mutant genotype*(T/T) at 194 bp. Lanes 5, 6, and 9 represented heterozygote genotype*(G/T) at 194, 101, and 93 bp, while Lane 17 showed homozygote genotype*(G/G) at 101, and 93 bp. Lane 11 identifies the DNA size marker (50‐1000Kbp).


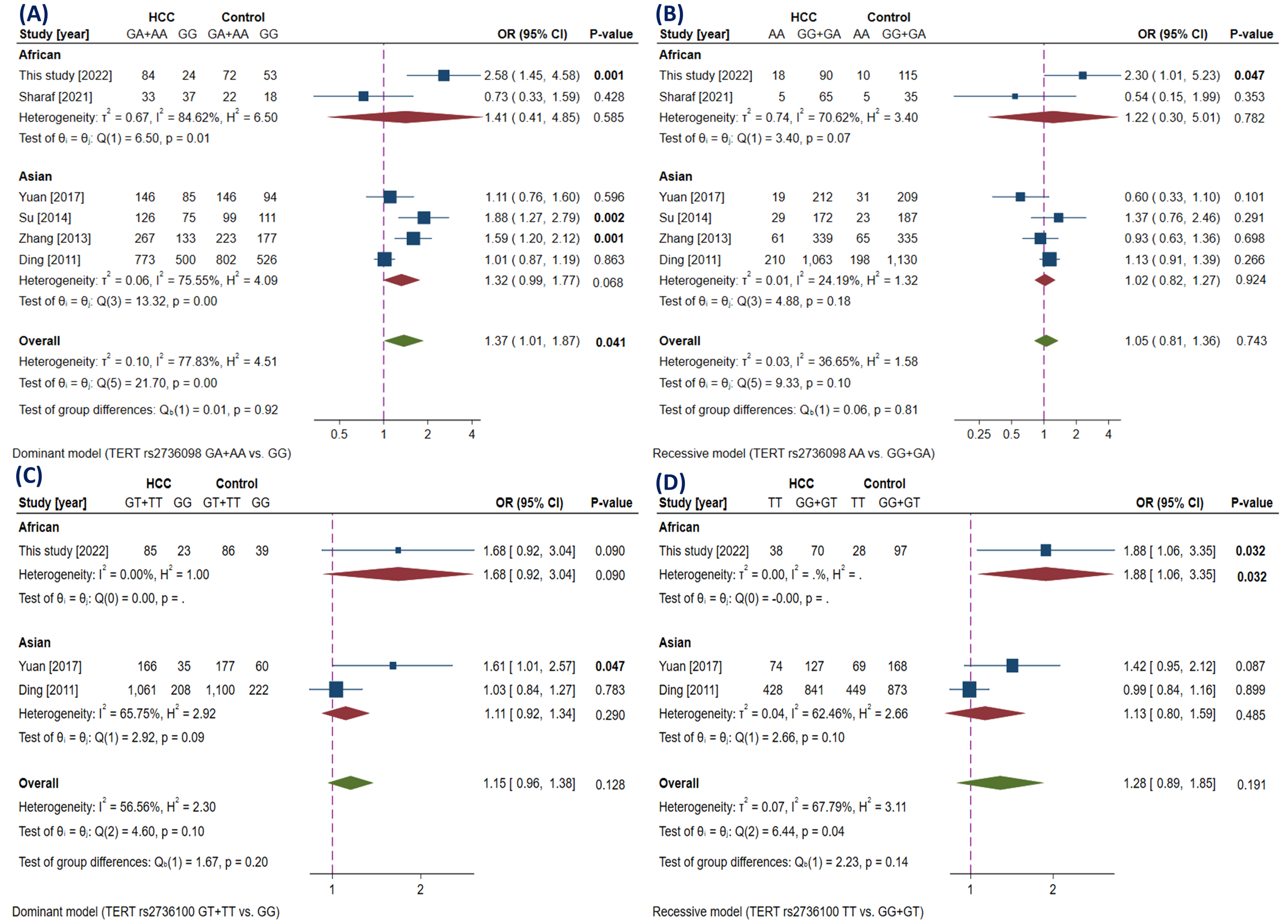


**Fig. S3. Forest plots of the pooled data for the association between *TERT (rs2736098 and rs2736100)* variants and HCC stratified by ethnicity under (A**) the dominant model of the *TERT (rs2736098, c.915G>A)* variant, **(B)** The recessive model of the *TERT (rs2736098, c.915G>A)* variant, **(C)** the dominant model of the *TERT (rs2736100; c.1574-3777G>T)* variant, and **(D)** the recessive model of the *TERT (rs2736100; c.1574-3777G>T)* variant.
